# Lymphopenia, Lymphopenia-Induced Proliferation, and Autoimmunity

**DOI:** 10.3390/ijms22084152

**Published:** 2021-04-16

**Authors:** Ting-Ting Sheu, Bor-Luen Chiang

**Affiliations:** 1 Department of Immunology, School of Medicine, College of Medicine, Tzu Chi University, Hualien 97004, Taiwan; 2 Department of Pediatrics, National Taiwan University Hospital, Taipei 10041, Taiwan; gicmbor@ntu.edu.tw; 3 Graduate Institute of Immunology, College of Medicine, National Taiwan University, Taipei 10051, Taiwan

**Keywords:** autoimmunity, immune aging, lymphopenia-induced proliferation, tolerance, Treg cells

## Abstract

Immune homeostasis is a tightly regulated system that is critical for defense against invasion by foreign pathogens and protection from self-reactivity for the survival of an individual. How the defects in this system might result in autoimmunity is discussed in this review. Reduced lymphocyte number, termed lymphopenia, can mediate lymphopenia-induced proliferation (LIP) to maintain peripheral lymphocyte numbers. LIP not only occurs in normal physiological conditions but also correlates with autoimmunity. Of note, lymphopenia is also a typical marker of immune aging, consistent with the fact that not only the autoimmunity increases in the elderly, but also autoimmune diseases (ADs) show characteristics of immune aging. Here, we discuss the types and rates of LIP in normal and autoimmune conditions, as well as the coronavirus disease 2019 in the context of LIP. Importantly, although the causative role of LIP has been demonstrated in the development of type 1 diabetes and rheumatoid arthritis, a two-hit model has suggested that the factors other than lymphopenia are required to mediate the loss of control over homeostasis to result in ADs. Interestingly, these factors may be, if not totally, related to the function/number of regulatory T cells which are key modulators to protect from self-reactivity. In this review, we summarize the important roles of lymphopenia/LIP and the Treg cells in various autoimmune conditions, thereby highlighting them as key therapeutic targets for autoimmunity treatments.

## 1. Introduction

A healthy and competent immune system must not only defend against infection but also protect against self-reactivity. To achieve this, it is critical that immune homeostasis maintains the T cell pool at a relatively constant number with a variety of specificities and tolerance to self-antigens [1,2].

In steady-state, large numbers of T cells are released from the thymus; these divide daily in the periphery and replace those that die by apoptosis [1]. Among T cell subsets, the most rapidly dividing population is of CD4^+^ regulatory T (Treg) cells, which suppress pathogenic, self-reactive cells that escape deletion during negative selection in the thymus [1]. Antigen-presenting dendritic cells (DCs) play the critical roles in the development, function, and homeostasis of Treg cells [3]. All these mechanisms contribute to immune homeostasis, in which self-tolerance is maintained. While the number of lymphocytes is low or reduced (lymphopenia) due to various triggers, a process termed lymphopenia-induced proliferation (LIP) could occur to respond to “fill-the-space” signals in the niche of secondary lymphoid organs [4,5,6]. However, this process might be associated with ADs such as type 1 diabetes (T1D) [7].

This article highlights the importance of T cell lymphopenia in the development of autoimmune diseases, as well as overviews LIP conditions to discuss the roles of loss of homeostatic control, focusing on Treg cells and negative costimulatory receptors during this LIP process.

## 2. Immune Homeostasis

Immune homeostasis is a tightly regulated system that is critical for defense against invasion by foreign pathogens and protects from self-reactivity for the survival of an individual. T lymphocytes play the critical roles in this regard and are the main focus of this review. Thymocytes are developed and produced from the thymus once they become mature T cells. In the periphery, the control of survival, proliferation, and death of T cells are also crucial to maintain T cell numbers. When the T cell number is decreased due to recent infection, treatment with certain cytotoxic medications, or other triggers, a recovery process of the T cell pool occurs through LIP.

Homeostasis of the T cell population could be mediated by two distinct modes of LIP: (i) slow homeostatic proliferation (HP) driven by low-affinity self-peptide/major histocompatibility complex (MHC) ligands and the cytokines interleukin (IL)-7 and IL-15 [4,5,8,9] and (ii) rapid spontaneous proliferation (SP) caused by high-affinity interactions with self-peptides or commensal bacterium-derived peptides presented by MHC molecules [10,11,12] as well as the cytokine IL-6 [13]. Of note, the complexity of T cell receptor (TCR) repertoire also regulate SP. Min et al. demonstrated that the presence of memory CD4^+^ T cells of broad diversity inhibited the proliferation and differentiation of transferred naïve CD4+ T cells; however, a memory population of similar number with limited diversity were not able to do so [14]. On the other hand, HP could be mediated by competition for cytokines, such as IL-7, by nonspecific T cells [15].

LIP might occur in both naïve and antigen-experienced memory T cells [16,17,18], and it has been shown that naïve T cells can acquire the characteristics of memory T cells during LIP [19,20,21,22]. Interestingly, Goldrath et al. demonstrated that when naïve CD8^+^ T cells transferred into sublethally irradiated (lymphopenic) syngeneic mice, they undergo slow HP and have a surface phenotype of memory CD8^+^ T cells (CD44^hi^Ly6C ^hi^CD122/IL-2Rβ^hi^) and are transiently able to produce interferon-gamma (IFN-γ) at early time points [21]. Even more interestingly, this HP-associated phenotypic conversion is reversible. After 20–30 days, the transferred cells begin to downregulate their expression of all three activation/memory markers. By 40–50 days after transfer, the majority of cells no longer express higher levels of these three surface markers. Similarly, after 12–31 days of transfer, the cells exhibit significant ability to produce IFN-γ whereas the ability to function as effectors in vitro diminishes by day 45 [21]. Of note, not only the naïve cells undergoing SP rapidly acquire the memory phenotypes, such as high CD44 expression and IFN-γ secretion [15,23], but also the SP of naïve cells produces cells that upregulate CD44 and IFN-γ expression to a greater degree than cells undergoing HP, thereby generating a majority of damaging autoreactive T cells [13,15,23]. It has been shown that if naïve T cells are transferred into syngeneic hosts with T-cell lymphopenia induced by irradiation, the majority of cells undergo HP, whereas only a small proportion of cells undergo SP [4,11]. In contrast, if transferred into lymphopenic RAG2^−/−^ mice, both HP and SP comparably occur within recipient mice [15]. The LIP of cells with the memory phenotype could be resulted from the SP of naïve or preexisting memory cells [4,10,11] as well as from HP regulated by IL-7/IL-15 because of a reduction in the number of naïve cells.

Interestingly, in addition to cytokines and the affinity between TCR and the self-peptide/self-MHC ligand, the rate of LIP of T cells depends on T cell subtype and age. In the case of naïve cells, the LIP might be faster for CD8^+^ T cells [13] than CD4^+^ T cells [15] as shown in the lymphopenic models generated by deletion of the *Rag2* gene. It has been shown that peripheral T cells from neonates proliferate more strongly compared to adult T cells, whereas it is not matched by increased expression of the activation marker CD44 [24].

In contrast, to prevent self-reactivity mediated by T lymphocytes, multiple tolerance mechanisms have been identified. Central tolerance is mediated by the removal of newly produced, strongly autoreactive lymphocytes (negative selection). Peripheral tolerance includes anergy (functional unresponsiveness), suppression by Treg cells, and deletion due to activation-induced cell death (AICD). It has been established that Treg cells with CD4^+^CD25^+^Foxp3^+^ phenotype are crucial for the maintenance of tolerance and originate from naturally occurring thymus-derived Treg cells (nTreg/tTreg) cells [25] and peripherally-induced Treg (iTreg/pTreg) cells generated from naïve cells upon antigen stimulation [26,27]. Mature self-antigen-reactive tTreg cells leave the thymus and enter peripheral tissues, where they can suppress the activation of other self-reactive T cells [28,29]. In addition, these two types of Treg cells mediate immune regulation through the production of IL-10, TGF-beta, and IL-35 or combinations of these proteins [30,31]. Other types of regulatory T cells originated from the periphery [1] comprise TGF-β-producing type 3 helper (Th3) cells [32] and IL-10-producing type 1 regulatory T (Tr1) cells [33]. Tr1 and Th3 cells are induced by DCs that have an activation status distinct from DCs that promote the differentiation of Th1 or Th2 cells.

Moreover, new subsets of Treg cells have been identified. The Foxp3^+^Bcl-6^+^CXCR5^+^ follicular regulatory T (Tfr) cells regulate the function of Tfh cells and the germinal center response [34,35,36]. IL-35-dependent regulatory cells demonstrate potent suppressive potential in several mouse disease models [31]. Other lymphocyte populations also contain regulatory cells. CD8^+^ Treg cells secrete either TGF-β or IL-10 [37,38]. Furthermore, antigen-activated CD8^+^ γδ T cells can prevent insulin-dependent diabetes in mice [39], and IL-10- and TGF-β-producing regulatory γδ T cells can suppress the antitumor activity of cytotoxic T lymphocytes (CTLs) and natural killer (NK) cells [40]. In addition, natural killer T (NKT) cells can secrete regulatory cytokines, including IL-10 [41]. Additionally, regulatory B (Breg) cells support immunological tolerance through the production of IL-10, IL-35, and TGF-β [42].

## 3. Lymphopenia in Physiological and Autoimmune Conditions

**Figure 1:**
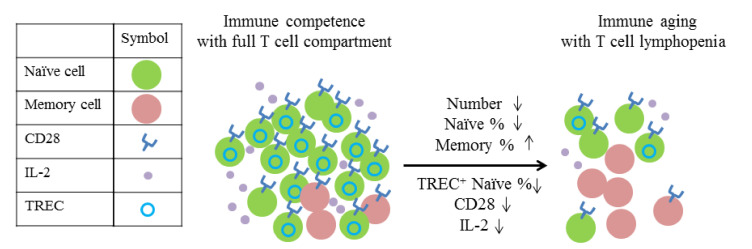
T cell compartment in different states.

Lymphopenia can occur in normal physiological conditions or be associated with abnormal pathogenic status such as autoimmune diseases. It has been shown that the number of T cells is very low in the peripheral immune system of neonatal mice. In CD4^+^ T cells, adult percentages are not reached until postnatal day 7 in the lymph nodes and day 15 in the spleen [43]. This lymphopenic state can drive LIP in neonates [44]. With aging, the thymic output in the elderly is reduced, leading to a decreased percentage of peripheral naïve T cells, which can lead to LIP [45]. Furthermore, in animals, it has been demonstrated that CD4^+^ and CD8^+^ T-cell lymphopenia exists in the spleens of aged Balb/c mice [46]. In addition, we demonstrated that in Balb/c mice, age was inversely correlated with the number of total and naïve CD4^+^ T cells, and advanced aging resulted in LIP, given that the percentage of proliferating cells is inversely correlated with the percentage of naïve cells [47]. In fact, T cell lymphopenia, especially a decline of naïve T cells, is a typical marker of immune aging (immunosenescence) in both humans and mice [46,47,48]. With aging, the recent thymic emigrants (RTEs) reduce, resulting from thymic involution. Newly generated T cells contain T-cell receptor excision circles (TRECs), which are small circles of DNA generated in T cells during their development in the thymus. TRECs are unable to replicate, and are diluted due to cell division, thereby are considered as a marker of newly generated T cell output. It is demonstrated that the percentage of TREC^+^ cells declines by more than 95% from 20 to 60 years of age [49,50]. Other than T cell lymphopenia, several typical markers of immune aging are identified, including the loss of CD28 expression, decreased IL-2 production and IL-2 receptor expression, and fewer naïve T cells containing TRECs [48] (Figure 1). It is well established that the loss of CD28 expression in CD4^+^ and CD8^+^ T cells in the elderly [51,52,53]. CD28 is a critical costimulatory signal for complementing TCR derived signals and promoting T cell proliferation and IL-2 production. The loss of CD28 expression is typically seen in T cells that have gone through multiple rounds of replication, and of note, clonal CD28^−^ T cells have been demonstrated to be autoreactive and resistant to apoptosis [54].

In addition, not only autoimmunity is increased in the elderly [55], but also lymphopenia is associated with various autoimmune conditions/diseases (Table 1). It has been demonstrated that lymphopenia occurs in primary immunodeficiency (PID) disorders, such as Omenn syndrome (OS) and Wiskott–Aldrich syndrome (WAS) [56,57,58,59], as well as common ADs; the onset of common ADs is secondary to a loss of tolerance to self-antigens. Interestingly, the associations between PIDs and ADs have been demonstrated. In fact, PIDs can be classified into four groups based on the frequency of association with ADs [60]. ADs are classified as organ-specific [61,62,63,64,65,66,67,68] or systemic [69,70,71,72,73,74,75,76,77,78,79,80] disorders (Table 1). Autoimmune conditions/diseases could be varied in the age of onset (e.g., early-onset or late-onset rheumatoid arthritis), single-gene traits [81], human leukocyte antigen (HLA) type [81,82,83,84,85] associated, or the trigger by viral infections [86,87] (Table 1). This phenomenon indicates that a common mechanism presented as lymphopenia may exist for the induction of autoimmunity. Of note, markers of immune aging have been documented in the ADs in humans [69,88,89,90,91,92,93,94,95,96] (Table 1), such as reduced percentage of TREC^+^ cells and/or lymphopenia, LIP in T cell subsets, and loss of CD28^+^ cells/accumulation of CD28^null^ cells in T cell subsets (Table 1).

### 3.1. Lymphopenia in Human Autoimmune Diseases

Lymphopenia has been found in T-cell deficiency PIDs, such as Omenn syndrome (OS) [57,58] and Wiskott–Aldrich syndrome (WAS) [59]. PIDs are genetic disorders that affect different parts of the immune system [103]. Patients with PIDs are with higher susceptibility to infections. In addition, they could be predisposed to immune dysregulation including malignancies [104,105,106], allergies [107], inflammation, and autoimmune diseases [108,109,110]. Interestingly, in a French national study, Fischer et al. demonstrate that, when analyzing age at onset, the probability of autoimmune or inflammatory manifestations occurred earlier in life up to around 20 years old is highest in patients with T-cell PIDs than in patients with innate and B-cell PIDs [111]. PIDs are monogenic disorders; therefore, their specific association with autoimmunity indicates the importance of a given gene in autoimmunity/tolerance.

OS is characterized by the infiltration of autologous and activated T cells into target organs such as the skin and gut [112]. This disorder could be affected by mutations in recombination-activating genes 1 and 2 (*RAG1* and *RAG2*), deoxyribonucleic acid (DNA) cross-link repair 1C (*DCLRE1C*), or DNA ligase 4 (*LIG4*) gene; these genes encode RAG1, RAG2, artemis, and DNA ligase 4, respectively. Of note, all these genes are involved in TCR rearrangement in the thymus, thereby contributing the deficiency of T cells. In addition, the TCR repertoire is severely restricted [113,114]. It has been found that a reduced number of Treg cells may play a critical role in inducing autoimmunity in OS given that the frequency of Treg cells is dramatically reduced in in the thymus and spleen of Rag2^R229Q/R229Q^ mice [115]. Reportedly, 70% of patients with WAS suffer from at least one of the following autoimmune disorders: hemolytic anemia, neutropenia, arthritis, skin vasculitis, glomerulonephritis, or inflammatory bowel disease [116]. This disorder is caused by mutations of a WAS protein (WASP). WASP is critical in cellular signaling to the actin cytoskeleton [117] to mediate the functions of multiple cell types in immune systems [118]. The immunopathogenic mechanisms in WAS might be linked to Treg function based on the following facts. First, investigations in WAS patients demonstrated that reduced suppressive function of Treg cells contribute to WAS autoimmunity [119]. Second, it has been shown that WASP is required for Treg cell-dependent suppression in vitro and in vivo [119,120]. Third, reduced production of IL-2, an important factor for the growth of CD4^+^CD25^+^FOXP3^+^ Treg cells, has been demonstrated in WAS patients [121].

In contrast to PID disorders, common ADs are of multifactorial origin with complex genetics involved [122,123,124,125,126,127,128]. The onset of these common ADs is secondary to the loss of self-tolerance and is directed against antigens present only in a particular organ (organ-specific) or in many organs and tissues (systemic) of the body, resulting in widespread tissue damage in the host [129] (Table 1).

Of note, T cell lymphopenia is a distinct characteristic in the ADs associated with T-cell PID disorders such as WAS [130,131]; however, despite its association with a variety of ADs as shown in Table 1, T cell lymphopenia may not be present in patients who are suffering from ADs [132,133] due to enduring LIP and/or the concomitant occurrence of other lymphoproliferative conditions such as lymphomas [134,135]. It has been shown that this concomitant occurrence of lymphoproliferative conditions might be linked to treatment with methotrexate (MTX), a disease-modifying antirheumatic drug (DMARD) [136,137,138]. Viral infections, including HIV [139,140], EBV [140,141], and HTLV-1 [142,143], may play critical roles in the development of both ADs and lymphoproliferative conditions. The possible roles of viral infections in LIP, autoimmunity, and lymphoproliferative conditions are discussed in Section 6.3 with focus on EBV since it has been shown to be associated with many ADs (Table 1).

Additionally, marked lymphopenia is associated with severe cases of the coronavirus disease 2019 (COVID-19) pandemic and has been used to predict disease severity [144,145,146] in COVID-19 patients infected with severe acute respiratory syndrome coronavirus 2 (SARS-CoV-2). It has been demonstrated that the lymphocyte number in COVID-19 patients gradually decreased as the disease progressed. In addition, older patients with lower lymphocyte and platelet counts were at higher risk of severe disease and increased duration of hospitalization [147]. This may be because SARS-CoV-2 invades progenitor/stem cells via angiotensin-converting enzyme 2 (ACE2) with its spike protein [148]. Lymphopenia occurs especially in T cells and NK cells [149]. The numbers of both CD4^+^ T cells and CD8^+^ T cells in patients with COVID-19 were lower than those in healthy donors, and the decrease in Th cells was more pronounced in severe cases [149]. Of note, several autoimmune disorders have been demonstrated in COVID-19 patients infected with SARS-CoV-2. These include cutaneous rashes and vasculitis, autoimmune cytopenia, antiphospholipid syndrome, central or peripheral neuropathy, myositis, and myocarditis [150].

### 3.2. Lymphopenia in Autoimmune-Prone Animal Models

In animal models, it has also been shown that lymphopenia may exist in PID disorders (e.g., OS and WAS) [97,98,99], organ-specific [7,100,101] (e.g., T1D), or systemic [102] (e.g., rheumatoid arthritis [RA]) ADs (Table 1). It has been shown that lymphopenia is associated with two animal models for OS. The memory mutant (MM) mouse exhibits a spontaneous mutation in *Rag1* (R972Q), which was also found in an OS patient [97]. The Rag2^R229Q/R229Q^ mouse exhibits a hypomorphic R229Q mutation in *Rag2* found in some OS patients [115]. Rag2^R229Q/R229Q^ mice have dramatically reduced frequency of Treg cells in the thymus and spleen, suggesting a reduced number of Treg cells may play a role in inducing autoimmunity in OS [115]. In addition, WASP-deficient mice are lymphopenic [98,99], and most of these mice develop colitis [98]. Investigations in WASP-deficient mice demonstrated that reduced suppressive function of Treg cells contribute to WAS autoimmunity [119]. Furthermore, lymphopenia is also found in animal models of nonobese diabetic (NOD) mice and K/BxN mice, which are animal models of T1D and RA, respectively [7,102].

## 4. LIP Is Associated with Autoimmunity in Both Animal Models and Humans

Interestingly, LIP not only occurs in normal physiological conditions as mentioned above but also correlates with autoimmunity in both humans and animal models (Table 1 and Table 2). This might be because the lymphopenic condition favors the production of self-reactive T cells through LIP and promotes autoimmunity based on the following findings. First, self-peptide/self-MHC can drive LIP [4,24]. Second, LIP is regulated by clonal competition [12,151]. It has been shown that T cells with higher TCR affinity can better compete for factors for cell survival and LIP [152]. Third, T cells undergoing LIP can acquire effector/memory function and thus contribute to the induction of autoimmunity [13]. The LIP-associated autoimmunity in animal models and patients is listed in Table 2. The induction of lymphopenia can be mediated by physiological condition [24,44], genetic alteration [4,13,14,15,97,102,153], irradiation [4,154,155], cytokine imbalance [7], immunosuppressive cytostatic drugs [156,157], impaired thymic function [49], or viral infections [147,149,150,158]. The mechanisms of LIP involving cytokines and TCR affinity are mainly demonstrated by murine models (Table 2).

### 4.1. LIP-Associated Autoimmunity in Animal Models

Genetic alterations mediated by transgenes or mutations may mediate lymphopenic conditions. A *Rag*-mutant mouse model of human OS exhibits lymphopenia, LIP, and activated CD4^+^ cells [97]. Furthermore, when purified CD8^+^ T cells were transferred into lymphopenic *Rag*-deficient mice, both slow HP and rapid SP were found as well as colitis. This rapid SP of naive CD8^+^ T cells caused colitis through the induction of pathogenic cytokine-producing Th17 cells and memory-type cells characterized by CD44 expression [13]. In addition, the LIP of transferred T cells in *Rag1*-mutant mice is enhanced in the absence of peripheral autoimmune regulator (AIRE) and induces colitis because of the decreased proliferation of Foxp3^+^CD4^+^ Treg cells [159]. Moreover, the importance of LIP in induction of autoimmunity has been demonstrated in an animal model of retinal autoimmune disease (experimental autoimmune uveoretinitis, EAU) [160]. In this study, a CD4^+^ β-galactosidase (βgal)-specific TCR transgenic mouse line (βgalTCR) and transgenic mice that are Rag^−/−^ and/or expressed βgal in retinal photoreceptor cells (arrβgal mice) were used. In the recipient mice, the lymphopenic condition was ensured by Rag mutation (Rag^−/−^). McPherson et al. showed that higher incidence and severity of EAU could be induced by transferring naïve, CD25-depleted βgal TCR T cells into lymphopenic arrβgal × Rag^−/−^ double transgenic recipient mice, compared to that induced by activated CD25-depleted βgal TCR T cells or in arrβgal transgenic recipient mice. It was also shown that LIP occurred and resulted in EAU in this experimental condition. These results suggest that lymphopenia/LIP plays a promoting role whereas regulatory T cells in the transferred T cells and in the recipient mice play inhibitory roles in the development of EAU [160].

Irradiation could also result in a lymphopenic state. It has been shown that when transferred into lymphopenic mice induced by irradiation, protein tyrosine phosphatase N2 (PTPN2)-deficient CD8^+^ T cells undergo rapid LIP and acquire the phenotypes of antigen-experienced effector T cells. PTPN2 is an important regulator of TCR signaling through dephosphorylation and inactivation of the Src family protein tyrosine kinases Lck and Fyn. The enhanced LIP, due to increased TCR-dependent but not IL-7-dependent responses, results in a skewed TCR repertoire and the development of autoimmunity, as indicated by increased hepatic lymphocytic infiltrates accompanying liver damage and production of antinuclear antibodies in sera [155]. Furthermore, it has been demonstrated that the LIP of naïve T cells in lymphopenic *Rag1*^−/−^ mice or irradiated mice is further enhanced by the deletion of *Tgfbr2*, the gene of TGF-βRII. It was found that signaling from TGF-β directly to naïve T cells inhibited their rapid proliferation, acquisition of effector functions, and autoimmunity in *Rag1*^−/−^ recipient mice [161,162].

Induction of lymphopenia by the drug cyclophosphamide (CTX) may regulate LIP, as demonstrated in an autoimmune diabetes model using the rat insulin promoter (RIP)-glycoprotein (GP)-transgenic mouse. In RIP-GP mice, the expression of the lymphocytic choriomeningitis virus (LCMV) glycoprotein in pancreatic β-cells is driven under the control of the RIP. After the adoptive transfer of activated CD4^+^ T cells specific for the I-A^b^-restricted LCMV GP61–80 epitope (Smarta), RIP-GP mice with a polyclonal CD8 TCR repertoire developed diabetes with a 20% onset rate. However, the onset rate of diabetes reached 100% when lymphodepletion was performed through CTX administration before the transfer of activated Smarta CD4^+^ T cells [156]. Therefore, the lymphopenic state in RIP-GP mice may contribute to the LIP of autoreactive CD8^+^ T cells.

### 4.2. LIP-Associated Autoimmunity in Humans

In humans, increased LIP of T cells has been demonstrated as a mechanism for the production of oligoclonal T cell repertoires in OS and WAS patients [58,163]. In OS, the TCR repertoire is severely restricted [113,114] and is characterized with large oligoclonal expansions of leaky TCR specificities. In addition, the pathology of OS has been linked to the presence of autologous T cells, which are mainly activated memory (CD25^+^HLA-DR^+^CD45RO^+^) cells [58,114,164]. This phenomenon is consistent with the concept that lymphopenia promotes the proliferation of cells with higher self-affinity, thereby promoting the accumulation of autoreactive T cells with memory phenotype and skewing the TCR repertoire toward autoimmunity.

Furthermore, it has been shown that a decline in the percentage of TREC^+^ T cells in RA patients, which occurred as a consequence of thymic involution and compensatory lymphopenia-induced proliferation in the periphery. LIP has been associated with clinical RA [74]. This may be because increased LIP could lead to TCR repertoire contraction and an increase in autoreactive T cell pool due to competition for self-peptide/self-MHC and cytokines, thereby predisposing the development of autoimmune diseases such as RA [74]. During this process of compensatory proliferation of post-thymic T cells, T cells with premature aging characteristics, including loss of cell surface receptor CD28, shortened telomeres, and production of high levels of IFN-γ, were accumulated. These premature aging T cells contributed autoreactivity and tissue damage. Premature telomere shortening in RA patients occurred in both CD4^+^ and CD8^+^ T cells [49]. In addition, it has been demonstrated that in primary Sjögren’s syndrome (pSS), lymphopenia mainly occurred in naïve CD4^+^ T cells. These naïve CD4^+^ T cells exhibited characteristics of aging such as shortened telomeres and reduced levels of TRECs, suggesting these naïve CD4^+^ T cells have gone through extensive post-thymic proliferation [95]. In patients of Hashimoto’s thyroiditis (HT), lymphopenia was noted especially in CD8^+^ T populations. Memory (CD45RO^+^) T cells were increased in both CD4^+^ and CD8^+^ T cells [90]. It has been shown that naïve (CD45RA^+^) CD4^+^ T cells showed lower TRECs numbers compared to healthy controls [90]. Although relative telomere length (RTL) was not significantly different between healthy controls and HT, a trend to shorter RTL was observed in naïve CD4^+^ T cells of HT patients. In addition, increased percentage of CD28^null^ cells was observed in T cells. Therefore, reduction of TRECs may be mostly caused by peripheral compensatory proliferation of naïve CD4^+^ T cells in HT. Nevertheless, whether other mechanisms by which reduction of TRECs existing remains to be examined, such as switching from naïve to memory cells, apoptosis of naive T cells or distribution to secondary lymphoid organs or the thyroid gland. Of note, the loss of CD28 expression was associated with cytomegalovirus (CMV)-seropositivity in HT patients [90].

It has been demonstrated that after one year of islet transplantation for T1D therapy, many patients were no longer insulin-independent and developed the autoimmune disease over time. These patients demonstrated both T cell and B cell lymphopenia due to the immunosuppressive drugs administered as part of the therapeutic protocol [157]. It is known that the immunosuppressive drugs used in this study, tacrolimus (FK506) and rapamycin, inhibit T cell signaling and thereby block the production of IL-2, a growth factor for T cells. This lymphopenia-induced T cell proliferation was associated with the production of more glutamate acid decarboxylase (GAD)-specific autoreactive T cells (β-cell reactive) and pancreatic islet destruction [157].

In addition, immune reconstitution inflammatory syndrome (IRIS) associated with HIV infection is the most well-defined lymphopenia-associated autoimmune disorder in humans. This clinical condition demonstrates the potent relationship between lymphopenia and organ-specific autoimmunity. Highly active antiretroviral therapy (HAART) leads to rapid increases in CD4^+^ T cell numbers in HIV-infected patients. The first increase in CD4^+^ T cell numbers could be observed days to weeks after the initiation of HAART. During the subsequent weeks and months, the efficiency of LIP is improved and patients show substantially increased CD4^+^ T cell numbers. IRIS occurs early during the rapid rise in CD4^+^ T cell numbers via LIP in HIV-infected patients with a history of viral infection or infection-associated inflammation that is well-controlled at the start of HAART. During the rapid rise in CD4^+^ T cell numbers, inflammation in the previous infection site recurs and the patients develop a recurrence of previous symptoms, most often without evidence of reinfection. Therefore, inflammation appears to be critical because of increased serum IL-6 levels and polymorphisms of IL-6, TNF-α, and IL-12 [165], and/or high-level inflammation in the tissue when immune reconstitution is initiated, predisposing to IRIS.

Moreover, as mentioned in Section 3, marked lymphopenia is associated with severe cases of COVID-19, and it was found that the proliferation of non-naïve CD4^+^ and non-naïve CD8^+^ T cells was significantly higher in COVID-19 patients than in healthy donors or recovered donors [166]. In the case of CD4^+^ T cells, the increased proliferation is mainly found in the effector memory population and circulating Tfh cells [166]. Therefore, the rapid reduction in lymphocytes may be critical in the pathogenesis through the accumulation of effector memory cells and contribute to severe COVID-19. In addition to lymphopenia, other markers involving immune aging have been demonstrated in severe and extremely severe COVID-19 patients including decreased CD28 expression and increased CD45RO (memory phenotype) expression on CD4^+^ and CD8^+^ T cells [167].

### 4.3. Setting in Which LIP Causes Autoimmune Diseases

LIP is not only associated with autoimmunity but has also been shown to play a causative role in the development of autoimmunity in animal models, including organ-specific and systemic autoimmune diseases. It is known that adhesion and degranulation-promoting adapter protein (ADAP) regulates positive selection, and ADAP deficiency results in a decreased thymic output. Another model for T1D, ADAP-deficient mice bred to the BDC2.5 TCR (pancreatic islet antigen-specific) transgenic mouse also exhibits lymphopenia, increased LIP, and diabetes. The transfer of either leukocytes or purified T cells into these lymphopenic mice resulted in reduced LIP and decreased diabetes incidence [153].

In addition, it has been demonstrated that the LIP of T cells occurs and plays a causative role in the development of T1D in NOD mice [7]. NOD mice exhibit CD4^+^ T-cell lymphopenia. The lymphopenic environment in NOD mice promotes the LIP of transferred pancreatic β cell-specific TCR transgenic NOD T cells. This increased T cell proliferation correlates with lymphocyte infiltration into the pancreatic islets. NOD mice with the greatest fraction of T cells proliferating in the lymphoid organs exhibited the greatest fraction of islets with insulitis. Immunization with complete Freund’s adjuvant (CFA), which is composed of immune-activating mycobacterial cell wall components, results in increased T cell numbers, decreased LIP of pathogenic β cell-specific T cells, and protection from T1D development. It was found that the production of cytokine IL-21 and a subsequent IL-21 receptor (IL-21R) expression on the surface of T cells increased in NOD mice compared to their congenic C57BL/6.Idd3.NOD mice. In contrast, it has been shown that NOD mice with IL-21R deficiency are protected from disease and demonstrate increased lymphocyte numbers and decreased HP. These data suggest that IL-21 promotes lymphopenia, HP, and autoimmunity [168]. Another T1D animal model, ADAP-deficient BDC2.5 TCR transgenic mice (pancreatic islet antigen-specific), also demonstrated that LIP causes autoimmunity as mentioned above [153]. Interestingly, by using the same model, NOD mice, we have previously demonstrated the presence of premature CD4^+^ T cell aging and its contribution to LIP of memory cells [47].

K/BxN mice (a cross between KRN TCR transgenic mice on a C57BL/6 background (K/B) and NOD mice) are a murine model of spontaneous RA. These mice are lymphopenic, but the resulting LIP of T cells can be prevented by the restoration of cell numbers [102]. During the preclinical phase of the disease, K/BxN mice exhibit CD4^+^ T-cell lymphopenia, which is followed by a compensatory proliferation of these cells during the early clinical phase. The majority of CD4^+^ T cells acquired a memory phenotype (CD44^high^CD62L^low^CD25^−^), which is a hallmark of lymphopenia-induced proliferating cells. K/BxN mice transferred with syngeneic T cells did not develop arthritis. This protective effect was associated with decreased proliferation of recipient-derived CD4^+^ T cells [102].

Furthermore, it has been shown that under lymphopenic conditions induced by irradiation, mice with a mutation in the gp130 IL-6 receptor subunit (F759) in nonhematopoietic cells developed an RA-like joint disease. These mice exhibited increased CD4^+^ T cell proliferation, and the disease is dependent on CD4^+^ T cells, given that mice lacking CD4^+^ T cells do not develop the disease. The F759 mutation resulted in greater proliferation caused by IL-7 through enhanced gp130-mediated STAT3 signaling, thus leading to increased memory/activated cells and disease. In contrast, providing CD4^+^ T cells prevents LIP and suppresses disease [154].

## 5. Lymphopenia Alone Is Not Sufficient to Induce Autoimmunity

Although autoimmunity is strongly associated with LIP as mentioned above, lymphopenia or LIP alone is not sufficient to cause autoimmune diseases. For example, humans with a lymphopenic state are found in diverse clinical conditions, such as congenital and acquired immunodeficiency syndromes, following autologous and allogeneic stem cell transplantation, antibody-depleting therapy to prevent or treat graft rejection, and cytotoxic chemotherapy for cancer [169]. These lymphopenic humans almost universally exhibit LIP, but most of them do not develop clinical evidence of autoimmunity.

In addition, neonatal mice are nearly devoid of peripheral T cells [24]. This lymphopenic state drives LIP without causing the development of autoimmunity [170]. In fact, only a minority of thymic emigrants undergo LIP in neonates. This is probably due to the presence of T cells with a Treg phenotype that is already found on day 3 after birth [171]. The LIP in neonatal mice leads to autoimmune diseases only if they are AIRE deficient. Interestingly, it has been shown that, during the perinatal period, AIRE promoted the generation of a distinct population of FOXP3^+^CD4^+^ Treg cells. These Treg cells stably existed in adult mice and played a role in maintaining self-tolerance to protect against the autoimmunity typical of AIRE knockout mice [172]. The AIRE expression in the perinatal age window is important since it is necessary and sufficient to avoid autoimmunity characteristic of AIRE-deficient mice [173]. PID patients with mutations in AIRE suffer from the autoimmune disease APECED (autoimmune polyendocrinopathy candidiasis ectodermal dystrophy) [174]. In addition, it was found that independent of the type of TCR stimulation, up to 70% of neonatal CD4^+^Foxp3^−^ T cells became CD4^+^Foxp3^+^ Treg cells, whereas less than 10% of adult CD4^+^Foxp3^−^ T cells became CD4^+^Foxp3^+^ Treg cells under the same conditions [175].

A two-hit model of autoimmunity states that in the presence of a trigger, as exemplified by lymphopenia, the loss of control over homeostasis may result in the onset of autoimmunity [158,161,162]. This two-hit model of autoimmunity is supported by several lines of evidence. For example, it has been shown that, in an animal colitis model, the transfer of T cells with naive phenotype (CD45RB^hi^) into lymphopenic severe combined immune deficiency (SCID) mice induces autoimmune colitis, whereas the transfer of T cells with memory phenotype (CD45RB^lo^) or mixed populations of CD45RB^hi^ plus CD45RB^lo^ T cells does not [176]. It has been shown that the deficiency in CD4^+^CD25^+^ regulatory cells is important because SCID mice transferred with CD4^+^CD25^−^ T cells also developed colitis, and this autoimmunity could be prevented by providing CD4^+^CD25^+^ cells within 10 days of CD4^+^CD25^−^ T cell transfer [177].

In addition, it has been shown that neonatal mice with thymectomy treatment developed autoimmune gastritis at high frequencies because of T cell reactivity developed recognizing the H/K ATPase self-antigen [178]. Similar to the models of colitis mentioned above, neonatal thymectomy in mice also leads to overall T cell lymphopenia with a preferential depletion of CD4^+^CD25^+^ Treg cells. This is because mice are lymphopenic at birth and undergo LIP in RTEs with high rates, which serves to effectively seed the peripheral T cell compartment [44]. It has been demonstrated that RTEs are the preferential precursors of Treg cells differentiated in the periphery [179].

Furthermore, in neonate physiological lymphopenia, induction of loss of homeostatic control by deletion of the *Tgfbr2* gene at an early stage results in the onset of autoimmunity and death before five weeks of age [180]. Histological analysis demonstrated severe infiltration of leukocytes into multiple tissues/organs including the stomach, lung, liver, pancreatic islets, and thyroid gland [180]. However, if deletion of the *Tgfbr2* gene is controlled to occur at a later stage, then there will be no development of autoimmunity in neonate mice even in the presence of lymphopenia [162].

In addition, as mentioned previously, IRIS occurs early during the rapid rise in CD4^+^ T cell numbers that occur via LIP in human immunodeficiency virus (HIV)-infected patients. Nevertheless, if T cell numbers are very low, autoimmune disease is not observed, suggesting lymphopenia alone is not able to cause autoimmunity.

## 6. The Loss of Control over Homeostasis

### 6.1. The Relationship between the Loss of Control over Homeostasis and the Function/Number of Treg Cells

**Figure 2:**
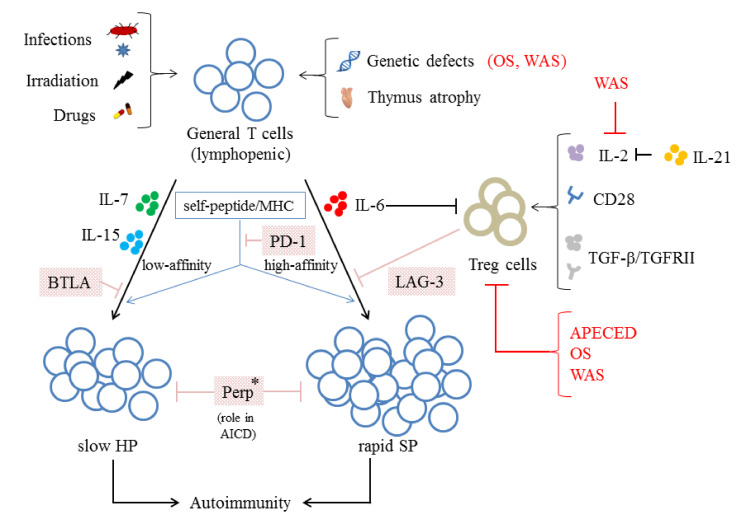
The induction, types and regulation mechanisms of LIP. Several triggers have been shown to induce lymphopenia including infection, irradiation, drugs, genetic defects, and thymus atrophy. LIP could occur via two pathways: slow homeostatic proliferation (HP) and rapid spontaneous proliferation (SP). HP is mediated by cytokines (IL-7 and/or IL-15) and interaction with self-peptide/MHC with low affinity. On the other hand, SP is regulated by cytokine IL-6 and interaction with self-peptide/MHC with high affinity. Treg cells might inhibit SP through LAG-3. Treg cells could be regulated by the presence of CD28, IL-2 production, and the TGF-β/TGFRII pathway; IL-21 could counteract Tregs function by inhibiting IL-2 production. IL-6 inhibits Treg cell function and expansion. PD-1 inhibits the LIP of T cells through its effect on peptide/MHC-II. BTLA negatively regulates the HP. Perp inhibits the persistence of T cells undergoing LIP through its role in activation-induced cell death; it is not known whether it exhibits a general role in both forms of LIP or in a certain form of LIP (∗). The roles of PIDs in the pathways of LIP are indicated in red. Arrows and T-bars indicate positive and negative effects, respectively.

Interestingly, during the LIP-associated autoimmunity mentioned in the previous Section 5, the mechanisms regarding the loss of control over homeostasis are all linked to Treg cells. The deficiency in CD4^+^CD25^+^ regulatory cells is important for lymphopenic SCID mice to develop colitis after transfer with naive CD45RB^hi^ T cells [177]. TGF-βRII deficiency plays important roles in the development of autoimmunity in neonate mice [180]. Interestingly, the TGF-β/TGF-βRII signaling pathway has also been shown to be critical for both the development and function of Treg cells [181]. IL-21 plays a role in lymphopenia induction in NOD mice, and Attridge et al. have shown that IL-21 can counteract Treg suppression through inhibition of T cell IL-2 production [182]. It is well known that IL-2 induces and enhances the growth of Treg cells (CD4^+^CD25^+^FOXP3^+^), which selectively inhibits the SP form of LIP [23]. Furthermore, increased level of IL-6 is observed in IRIS. It is known that IL-6 inhibits Treg cell function [183] and Treg cell expansion [184]. IL-6 also induces Smad7, thereby inhibiting TGF-β activity [185] (Figure 2).

### 6.2. Other Mechanisms Negatively Regulate the LIP of T Cells

Several mechanisms have been demonstrated to negatively regulate the LIP of T cells. Programmed cell death protein 1 (PD-1) plays a role in inhibiting the LIP of T cells, and this effect is dependent on peptide/MHC-II but not IL-7 Ralpha signaling [186]. In addition, an MHC class II-binding CD4 homolog, lymphocyte activation gene 3 (LAG-3), regulates the LIP of T cells by both Treg-dependent and independent mechanisms [187]. B and T lymphocyte attenuator (BTLA) negatively regulates the HP of CD8^+^ T cells [188]. Perp (p53 apoptosis effector related to PMP-22) inhibits the persistence of CD4^+^ effector/memory T cells undergoing LIP through its role in AICD [189] (Figure 2).

### 6.3. The Possible Roles of Viral Infections in LIP, Autoimmunity, and Lymphoproliferative Conditions

**Figure 3:**
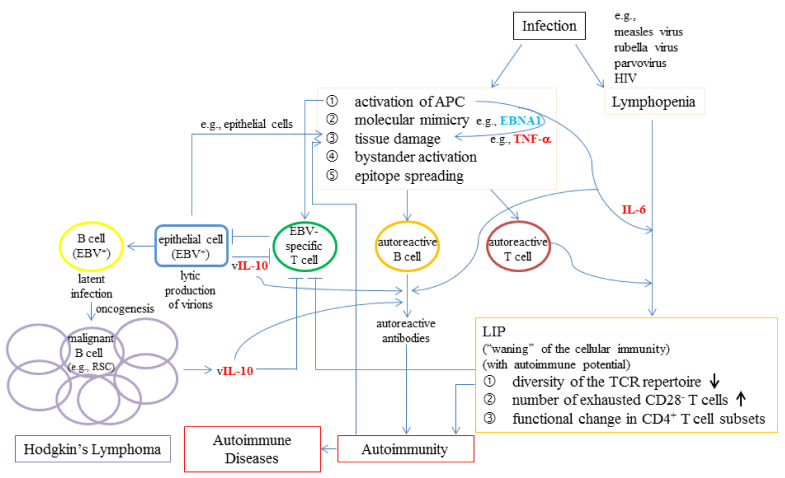
The possible roles of viral infections in LIP, autoimmunity, and lymphoproliferative conditions with focus on EBV. Infections could lead to lymphopenia and production of autoreactive T cells (brown circle) and B cells (orange circle) through a series of mechanisms. Under lymphopenic condition, IL-6 may drive LIP of autoreactive T cells. The LIP of autoreactive T cells and the production of autoantibodies increase autoimmunity that may further develop into autoimmune diseases. Cytokine TNF-α released from activated APCs could cause tissue damage. Activation of APCs by EBV infection results in the production of EBV-specific effector T cells such as cytotoxic T cells (green circle). EBV could infect both epithelial cells and B cells, although B cells are the primary targets. For illustration purpose only, it is shown that EBV virions produced from epithelial cells (blue rectangle) during the lytic phase of its life cycle could cause further damage of epithelial cells. EBV could also enter into a latent phase, during which the virus could remain silent in memory B cells (yellow circle). EBV has evolved various strategies to modulate the host immune response, such as production of viral IL-10 (vIL-10), which contribute to development of EBV^+^ B cell lymphomas, such as RSC positive Hodgkin’s lymphoma. Arrows and T-bars indicate positive and negative effects, respectively.

Infections may mediate LIP by several ways. First, lymphopenia is frequently associated with many viral infections, such as influenza virus [190,191], measles virus [192], rubella virus [193], parvovirus [194], HIV [195], and SARS-CoV-2 [149]. Second, activation of autoreactive T cells can be mediated by infections with bacteria or viruses through the following mechanisms [196]: (i) molecular mimicry, (ii) bystander activation after tissue damage caused by proinflammatory cytokines (such as TNF-α) which are released from antigen-presenting cells (APCs) after stimulation of Toll-like receptors (TLRs) and other pattern-recognition receptors (PRRs) [141], and (iii) epitope spreading to other autoreactive B cells after more tissue destruction by activated autoreactive T cells and inflammatory cytokines [196]. Third, proinflammatory cytokine IL-6 can further regulate autoimmunity by inducing LIP and autoantibody production, thereby establishing a vicious cycle (Figure 3). The enduring LIP process due to the presence of infections could lead to accumulation of cells with the following characteristics: loss of diversity of the TCR repertoire, increase in number of exhausted CD28^−^ T cells, and functional changes in CD4^+^ T cell subsets. These cells are with autoimmune potential yet are waning of the cellular immunity.

Taking EBV infection in RA as an example (Figure 3), molecular mimicry occurs between EBV and self-proteins such as: Epstein–Barr nuclear antigen 1 (EBNA1) and human cytokeratin; a sequence in the gp110 EBV glycoprotein and sequence QKRAA located in HLA-DRB1*0401, a susceptibility factor for RA [197]. In addition, during its lytic cycle, EBV can produce a human IL-10 homolog (vIL-10). Human IL-10 stimulates anti-inflammatory responses whereas vIL-10 is less efficient in this regard. Moreover, vIL-10 inhibits the increase of suppressors of inflammation induced by hIL-10 [198]. Furthermore, vIL-10 interferes with the activity of CD4^+^ Th cells [199], prevents an attack by nature killer cells [199], promotes proliferation/differentiation of B cells [200], and stimulates antibody production [200]. EBV is associated with cancers and lymphoproliferative conditions originated from both epithelial cells and hematopoietic cells such as Hodgkin’s lymphoma caused by Reed–Sternberg cells (RSCs) [201].

It is reasoned that EBV infection could enhance LIP of host autoreactive cells, thereby dampening the ability of cellular immunity to clear virus-infected host cells. This concept is supported by several studies. First, it has been demonstrated that EBV infection occurred in most Burkitt’s lymphoma (BL) cases in solid organ transplant recipients; the onset rate of BL was decreased in those patients who were EBV-seropositive at baseline compared to EBV-seronegative [202]. EBV is also associated with post-transplant lymphoproliferative disorder (PTLD). Second, it was found that decreased T-cell response to EBV gp110 glycoprotein, expressed in lytic cycle in RA, was correlated with inflammation and systemic involvement [197]. Third, very interestingly, it has been demonstrated the contribution of cytokines, TNF-α, IL-6, and IL-10, to the association of ADs with specific types of lymphoproliferative conditions based on evaluating the effect of single nucleotide polymorphism (SNP) of cytokines in the progression of lymphoma in concurrence with ADs [134].

In summary, from the perspective of viruses, turning the host’s immune system to continuously attack hosts themselves through LIP of host autoreactive cells and thus making it eventually exhausted to avoid being attacked, is an excellent strategy for their own long-term survival and eventual expansion at the expense of host health.

## 7. Conclusions

A healthy and competent immune system needs the maintenance of immune homeostasis with tolerogenic responses to self-reactive cells. If LIP endures, as opposed to returning to this steady-state homeostasis, it could lead to the development of ADs. The loss of homeostatic control might be linked to Treg cells and several coinhibitory receptors that can negatively regulate the LIP of T cells. Indeed, many single-gene traits linked to autoimmune diseases (e.g., AIRE and CTLA-4) play important roles in the production or function of Treg cells. Importantly, a variety of ADs are associated with lymphopenia, although susceptibility to individual autoimmune diseases might be linked to certain HLA genes and/or infection with viruses. Therefore, it is reasoned that the exploration of the mechanisms by which LIP is regulated may provide information for the development of both preventive and therapeutic strategies to conquer ADs.

## Figures and Tables

**Table 1:** Lymphopenia-associated disorders in humans.

Human Disorders with Autoimmune Manifestation	Lymphopenia	Disease-Associated Factor			Animal Model with Lymphopenia	The StudyInvolves Marker(s) of Immune Aging
		Single-Gene Traits [81]	HLA [81]	Virus Infection [86]		
*Primary immunodeficiency disorder*						
Omenn syndrome	[56,57,58]	*RAG1/2,* *DCLRE1C*, *LIG4*			[97]	
Wiskott–Aldrich syndrome	[59]	*WASP*			[98,99]	
*Common ADs secondary to a loss of self-tolerance*						
**Organ-specific disorder**						
Type 1 diabetes	[61]	*CTLA-4, INS*	DQ2, DQ8, DR3, DR4	Coxsackievirus B4,CMV, mumps virus, and rubella virus	[7]	[88] ^c^, [47] ^a,b,c^ (mouse model)
Multiple sclerosis	[62]		DR2	EBV, measles virus	[100]	[89] ^a,c^
Graves’ disease	[63]	*CTLA-4*	DR3	Coxsackie B virus, retrovirus, and HCV		
Myasthenia gravis	[64,65]		DR3	HCV, HSV		
Hashimoto’s thyroiditis	[66]		DR5	HTLV-1, enterovirus, rubella virus, mumps virus, HSV, EBV, and parvovirus	[101]	[90] ^a,b,c^
Primary biliary cirrhosis	[67]			HCV		[91] ^c^
Autoimmune hemolytic anemia	[68]			EBV, VZV (mouse model) [87]		[92] ^c^
**Systemic disorder**						
Juvenile idiopathic arthritis	[69]					[69] ^a,b^
Juvenile rheumatoid arthritis	[70]					
Systemic lupus erythematous	[71,72]	*C1q*	DR3	EBV		[93] ^a,c^
Rheumatoid arthritis	[73,74]		DR4	EBV, HCV	[102]	[89] ^a,c^, [94] ^a,b,c^
Dermatomyositis	[75,76]		B*08:01 [82]			
Polymyositis	[76]		DRB1*03:01 [82]			
Primary Sjogren’s syndrome	[73,77]		B8, Dw3 [83]	EBV		[95] ^a,b^
Systemic sclerosis	[78,79]		DQ5, DQ7 [84]			[96] ^c^
Crohn’s disease	[80]		DQ5 [85]			

^a^ Reduced in the percentage of TREC^+^ cells and/or lymphopenia in T cell subsets; ^b^ Lymphopenia-induced proliferation in T cell subsets; ^c^ Loss of CD28^+^ cells or accumulation of CD28^null^ cells in T cell subsets. Abbreviations: Rag-1/-2, recombination activating gene-1/-2; WASP, Wiskott–Aldrich syndrome protein; CTLA-4, cytotoxic T-lymphocyte-associated protein 4; INS, insulin; C1q, complement component 1q; HLA, human leukocyte antigen; CMV, cytomegalovirus; EBV, Epstein–Barr virus; HCV, hepatitis C virus; HSV, herpes simplex virus; HTLV-1, human T-lymphotropic virus 1; VZV, varicella-zoster virus.

**Table 2:** LIP and its presence in physiological and autoimmune conditions.

Investigator, Year	Lymphopenia Type	Model, Transferred Donor	Findings ^a^	Reference
Le Campion, 2002	Physiologically-related	Neonate B6 mice, None	The LIP of peripheral (spleen) naïve T cells involves TCR interactions with self-peptide/self-MHC complexes Peripheral T cells from neonates proliferate more strongly compared to adult T cells, whereas it is not matched by increased expression of activation marker CD44	[24]
Min, 2003	Physiologically-related	Neonate B6 mice, Adult B6 peripheral LN CD4^+^ T cells	Neonates support LIP of CD4^+^ T cells isolated from adult peripheral lymph nodes (LNs) The LIP includes slow LIP (HP) and fast LIP (SP) HP: <7 times/16–18 days SP: ≥7 times/16–18 days Adult naïve T cells acquire memory phenotypes and functions especially through SP LIP is inhibited by the presence of both memory and naïve T cells SP: MHC II-TCR dependent; IL-7-independent	[44]
Ernst, 1999	Genetically-induced	TCRα^−^ mice, Whole B6. PL LN cells	SP of LN CD4^+^ T cells: ~1 time/day Autoimmunity: not determined	[4]
Min, 2004	Genetically-induced	TCR transgenic *Rag*^−/−^ mice, Polyclonal naïve T cells	The capacity of some polyclonal naïve T cells to SP upon transfer into a lymphopenic environment is not simply due to a deficiency in the numbers of lymphocytes Memory cells inhibit the SP of naïve cells TCR recognition of antigen is regulated by the TCR repertoire complexity Autoimmunity: not determined	[14]
Min, 2005	Genetically-induced	Rag2^−/−^ (B10.A background) mice, Naïve CD4^+^ T cells	HP: IL-7 dependent; ≤1–2 times/7 days SP: IL-7 independent; ~1 time/day The SP of naïve cells produces cells that upregulate CD44 and IFN-γ expression to a greater degree than cells undergoing HP Autoimmunity: not determined	[15]
Jang, 2006	Genetically-induced	K/BxN mice (a cross between KRN TCR transgenic mice on a C57BL/6 background (K/B) and NOD mice), None	Prevention of spontaneous arthritis is mediated by the inhibition of homeostatic expansion of autoreactive CD4^+^ T cells in the K/BxN mouse model	[102]
Khiong, 2007	Genetically-induced	Rag1 mutation (reduced activity) mice, None	The LIP of CD4^+^ T cells is involved in the pathogenesis of an Omenn syndrome murine model	[97]
Tajima, 2008	Genetically-induced	Rag2^−/−^ (C57BL/6 background) mice, Naïve CD8^+^ T cells	IL-6-dependent SP is required for the induction of colitis through IL-17-producing CD8^+^ T cells characterized by CD44 expression SP: induction of flora-specific colitis SP of CD8^+^ T cells: ≥2 times/day SP mainly occurs in mesenteric LN	[13]
Zou, 2008	Genetically-induced	ADAP-deficient mice bred to the BDC2.5 TCR transgenic mice, None	The presence of decreased thymic output and LIP The onset of type 1 diabetes The transfer of either leukocytes or purified T cells into ADAP-deficient mice leads to reduced LIP and decreased diabetes incidence	[153]
Ernst, 1999	Irradiation	600 cGy, Whole B6. PL LN cells	TCR/MHC interaction is critical in the LIP of CD4^+^ and CD8^+^ T cells HP of LN CD4^+^ T cells: ≤0.5 time/day SP of LN CD4^+^ T cells: ~1 time/day (a small proportion) Autoimmunity: not determined	[4]
Sawa, 2006	Irradiation	9.5 Gy on a mouse with Gp130 IL-6 receptor mutation (F759 mouse), None	Development of rheumatoid arthritis-like joint disease Increased CD4 T cell proliferation through enhanced gp130-mediated STAT3 signaling results in increased memory/activated cells LIP of CD4^+^ T cells is increased due to elevated production of IL-7 by non-hematopoietic cells as a result of IL-6 family cytokine-gp130-STAT3 signaling	[154]
Wiede, 2014	Irradiation	Sub-lethal irradiation (600–650 Gy), PTPN2-deficient naïve CD8^+^ T cells	PTPN2-deficient naïve CD8^+^ T cells undergo rapid LIP when transferred into irradiated lymphopenic mice and acquire the characteristics of antigen-experienced effector T cells The increased LIP response is TCR-dependent but not IL-7-dependent The LIP results in an altered TCR repertoire and the development of autoimmunity (increased hepatic lymphocytic infiltrates accompanying liver damage)	[155]
King, 2004	Cytokine-mediated	NOD mice, β cell antigen-specific 8.3-NOD CD8^+^ T cells	The LIP in secondary lymphoid organs correlates with the type and severity of islet infiltration and generates type 1 diabetes Include cells dividing >1 time/day	[7]
Calzascia, 2008	Immunosuppressive cytostatic drug-induced	Cyclophosphamide-induced lymphopenia in RIP-GP mice (a model of beta-islet cell self-reactivity), None	The physiological lymphopenia-associated production of IL-7 can profoundly promote the LIP of self-reactive clones in the presence of regulatory T cells Autoimmune diabetes rapidly ensued with CD4 help and the subsequent activation of CD8 T cells, which contributed to disease progression	[156]
Monti, 2008	Immunosuppressive drugs-induced	Administration of FK506 and rapamycin in islet transplantation patients, None	T cell loss after islet transplantation in patients with type 1 diabetes was associated with both increased serum concentrations of IL-7 and IL-15 and in vivo proliferation of memory CD45RO^+^ T cells, highly enriched in autoreactive GAD-specific T cell clones Immunosuppression with FK506 and rapamycin after transplantation resulted in a chronic LIP of T cells, which acquired effector function	[157]
Koetz, 2000	Impaired thymic function	Rheumatoid arthritis patients,None	The thymic output is decreased, which might result in increased LIP of CD4^+^ and CD8^+^ T cells with autoreactive T cells in the periphery The LIP is observed in naïve T cells as demonstrated by telomere shortening	[49]
Krupica, 2006	Virus infection	HIV-mediated reduction of CD4^+^ T cells in patients, None	HARRT improves the efficiency of LIP to reconstitute CD4^+^ T cells IRIS occurs during HARRT therapy	[158]

^a^ The rate of HP and/or SP was evaluated by the author of this study. Abbreviations: LIP, lymphopenia-induced proliferation; TCR, T cell receptor; MHC, major histocompatibility complex; CD44, cluster of differentiation 44; CD4, cluster of differentiation 4; HP, homeostatic proliferation; SP, spontaneous proliferation; IL-7, Interleukin-7; Rag, recombination activating gene; IL-6, Interleukin-6; IL-17, Interleukin-17; CD8, cluster of differentiation 8; ADAP, adhesion and degranulation-promoting adapter protein; cGy, centigray; Gy, gray; STAT3, signal transducer and activator of transcription 3; PTPN2, protein tyrosine phosphatase N2; NOD, non-obese diabetic; RIP-GP, rat insulin promoter-glycoprotein; FK506, tacrolimus; GAD, glutamate acid decarboxylase; IRIS, immune reconstitution inflammatory syndrome; HARRT, highly active antiretroviral therapy.

## Data Availability

Not applicable.

## Abbreviations

ACE2	angiotensin-converting enzyme 2
ADs	autoimmune diseases
ADAP	adhesion and degranulation-promoting adapter protein
AIRE	autoimmune regulator
APCs	antigen-presenting cells
APECED	autoimmune polyendocrinopathy candidiasis ectodermal dystrophy
Bcl-6	B-cell lymphoma 6
βgal	β-galactosidase
BL	Burkitt’s lymphoma
Breg	regulatory B cells
BTLA	B and T lymphocyte attenuator
CFA	complete Freund’s adjuvant
CMV	cytomegalovirus
COVID-19	coronavirus disease 2019
CTLs	cytotoxic T lymphocytes
CTLA-4	cytotoxic T-lymphocyte-associated protein 4
CTX	cyclophosphamide
CVID	common variable immunodeficiency
CXCR5	C-X-C chemokine receptor type 5
DCs	dendritic cells
DCLRE1C	deoxyribonucleic acid cross-link repair 1C
DMARD	disease-modifying anti-rheumatic drug
EAU	experimental autoimmune uveoretinitis
EBNA1	Epstein–Barr nuclear antigen 1
EBV	Epstein–Barr virus
F759	mice with a mutation in the gp130 IL-6 receptor subunit
FK506	tacrolimus
FoxP3	forkhead box protein transcription factor P3
GAD	glutamate acid decarboxylase
HAART	highly active antiretroviral therapy
HIV	human immunodeficiency virus
HLA	human leukocyte antigen
HP	homeostatic proliferation
HT	Hashimoto’s thyroiditis
HTLV-1	Human T-lymphotropic virus 1
IFN-γ	interferon-gamma
IL	Interleukin
IL-2R	IL-2 receptor
IL-21R	IL-21 receptor
INS	insulin
IRIS	immune reconstitution inflammatory syndrome
iTreg/pTreg	peripherally-induced Treg cells
K/BxN	a cross between KRN TCR transgenic mice on a C57BL/6 background (K/B) and NOD mice
LAG-3	lymphocyte activation gene 3
LCMV	lymphocytic choriomeningitis virus
LIG4	ligase 4
LIP	lymphopenia-induced proliferation
LNs	lymph nodes
MHC	major histocompatibility complex class
MM	memory mutant
MTX	methotrexate
NK	natural killer cells
NKT	natural killer T cells
NOD	non-obese diabetic
nTreg/tTreg	naturally occurring thymus-derived Treg cells
OS	Omenn syndrome
PD-1	programmed cell death protein 1
Perp	p53 apoptosis effector related to PMP-22
PID	primary immunodeficiency
PIDs	primary immunodeficiencies
PRRs	pattern-recognition receptors
pSS	primary Sjögren’s syndrome
PTLD	post-transplant lymphoproliferative disorder
PTPN2	protein tyrosine phosphatase N2
RA	rheumatoid arthritis
RAG1	recombination activating gene 1
RAG2	recombination activating gene 2
RIP-GP	rat insulin promoter-glycoprotein
RSCs	Reed-Sternberg cells
RTL	relative telomere length
RTEs	recent thymic emigrants
SARS-CoV-2	severe acute respiratory syndrome coronavirus 2
SCID	severe combined immune deficiency
SLE	systemic lupus erythematosus
SNP	single nucleotide polymorphism
SP	spontaneous proliferation
SS	Sjögren’s syndrome
STAT3	signal transducer and activator of transcription 3
T1D	type 1 diabetes
TCR	T cell receptor
Tfh	T follicular helper
Tfr	follicular regulatory T cells
TGF-β	transforming growth factor-beta
TGF-βRII	transforming growth factor-beta receptor 2
tgfbr2	transforming growth factor-beta receptor 2
Th1	type 1 helper cells
Th3	type 3 helper cells
Th17	type 17 helper cells
TLRs	Toll-like receptors
TNF-α	tumor necrosis factor-alpha
Tr1	type 1 regulatory T cells
TRECs	T-cell receptor excision circles
Tregs	regulatory T cells
WAS	Wiskott–Aldrich syndrome
WASP	WAS protein
